# A hypothesis on biological protection from space radiation through the use of new therapeutic gases as medical counter measures

**DOI:** 10.1186/2045-9912-2-8

**Published:** 2012-04-04

**Authors:** Michael P Schoenfeld, Rafat R Ansari, Atsunori Nakao, David Wink

**Affiliations:** 1 National Aeronautics and Space Administration Marshall Space Flight Center, Huntsville, Alabama, USA; 2 National Aeronautics and Space Administration John H Glenn Research Center, Cleveland, Ohio, USA; 3 Department of Surgery, University of Pittsburgh, Pittsburgh, PA, USA; 4 National Institute of Health, National Cancer Institute, Radiation Biology Branch, Bethesda, Maryland, USA

**Keywords:** space radiation, radiolysis, radiochemistry, radiation shielding, therapeutic medical gas, reactive oxygen species, oxidative stress, countermeasure

## Abstract

Radiation exposure to astronauts could be a significant obstacle for long duration manned space exploration because of current uncertainties regarding the extent of biological effects. Furthermore, concepts for protective shielding also pose a technically challenging issue due to the nature of cosmic radiation and current mass and power constraints with modern exploration technology. The concern regarding exposure to cosmic radiation is biological damage that is associated with increased oxidative stress. It is therefore important and would be enabling to mitigate and/or prevent oxidative stress prior to the development of clinical symptoms and disease. This paper hypothesizes a "systems biology" approach in which a combination of chemical and biological mitigation techniques are used conjunctively. It proposes using new, therapeutic, medical gases as chemical radioprotectors for radical scavenging and as biological signaling molecules for management of the body's response to exposure. From reviewing radiochemistry of water, biological effects of CO, H_2_, NO, and H_2_S gas, and mechanisms of radiation biology, it can be concluded that this approach may have therapeutic potential for radiation exposure. Furthermore, it also appears to have similar potential for curtailing the pathogenesis of other diseases in which oxidative stress has been implicated including cardiovascular disease, cancer, chronic inflammatory disease, hypertension, ischemia/reperfusion (IR) injury, acute respiratory distress syndrome, Parkinson's and Alzheimer's disease, cataracts, and aging. We envision applying these therapies through inhalation of gas mixtures or ingestion of water with dissolved gases.

## The Challenge of Space Radiation

Galactic Cosmic Rays (GCR), solar energetic particles (SEP), and trapped energetic particles in a planetary magnetic field are natural sources of radiation in space. GCRs consist of highly energetic nuclei, predominately protons and He, but also trace amounts of C, O, Ne, Si, Ca, and Fe ions. Particle energies can range from 100 MeV to 10 GeV per nucleon [1]. Although the high charge and energy (HZE) nuclei are in trace amounts, they are still of concern because they can cause more damage than protons since they are more highly ionizing. As well, even though particle fluxes are typically low, they are chronic and can significantly increase with solar events [1]. Furthermore, GCRs and SEPs impinging on shielding material, atmosphere, or surface of a planet or satellite can produce secondary radiation, including energetic neutrons, from nuclear fragmentation of the primary ion and target atoms. This can introduce an additional component to the radiation field which makes shielding from HZE quite challenging and poses one of the principal unknowns in understanding the HZE effects with human tissue [2]. Furthermore, while our bodies do possess a natural repair mechanism, radiation with a high linear energy transfer (LET) rate, like space radiation, is attributed to be more likely to cause double strand breaks in DNA that are relatively more difficult for our natural repair mechanisms to fix correctly [3]. While a week or month of this radiation at the dose rates naturally present likely will not have serious consequences, several year durations in space could. The traditional paradigm for radiation protection is to minimize exposure time, maximize distance from radiation sources, and use shielding to attenuate and absorb radiation before it can deposit its energy in humans. In regards to minimizing exposure time, new propulsive technologies could reduce trip times but have yet to be developed and would not address the ability to remain at a location for long durations. It is impractical to maximize distance from cosmic radiation sources. In regards to shielding, aspects of attenuation by mass or deflection by magnetic fields or charge repulsion have been considered. Due to the phenomena of secondary radiation, shielding by other matter may require a significant amount of mass which could be impractical within current mass constraints in space systems. Due to the high energy of the space radiation, magnetic field and charge strengths required for deflection may be currently impractical because of mass and power constraints in modern space systems along with other system design implications. In short, shielding space radiation is seemingly quite challenging. However, advances in biochemistry may reveal some more tools for radiation protection [2].

## Parallels between Radiation Chemistry of Water & Radiobiology

Radiolysis is the decomposition of water from exposure to ionizing radiation. Radiation chemistry of water has been well studied since the onset of nuclear power production, as water has been the most often used coolant. Since mammalian cells are composed of about 80% water, it seemed natural that there exist similarities between radiation chemistry of water and radiation biology. It is these similarities from which analogues for radioprotective measures were inspired.

### Chain of Events Initiated by Chemically Reactive Species

Radiolysis in nuclear systems causes a chain of events that ultimately manifest into systematic problems like corrosion and gas generation. Ionizing radiation creates chemically reactive radicals H_3_O^+^, e^-^, H^+^, H, and OH by ionizing and/or breaking the bonds of water molecules. These radicals then initiate a chain of chemical reactions within the water which can result in the formation of molecular decomposition products such as H_2_, O_2_, HO_2 _and H_2_O_2_. BWR recirculation water contains oxygen and hydrogen peroxide in the concentration range from 100 to 300 ppb, and about 10 ppb of dissolved hydrogen (less than stoichiometric ratio of 8 to 1) [4]. These oxidizing species alter the water composition and therefore its electrochemical character which facilitates the manifestation of problems like corrosion or gas generation. As such, the nature in which systematic problems develop can be viewed as stemming from a chain of events that are initiated by ionization and propagated by a scheme of chemical reactions with the net result or outcome depending upon the ensuing chemistry.

This scenario is similar in nature to a biological system and the pathogenesis of radiation related ailments and disease. Ionization of key biological molecules can lead to chemical reactions which transform these molecules. This alters their biochemical function and can result in changes of their biochemical properties. Modification of biochemical properties propagates from a cellular level to organ and systematic changes that ultimately manifest into clinical symptoms and ailments. Ionization of the molecules can be initiated both directly (by radiation) and indirectly (by free radicals and reactive oxygen species (ROS) created by radiolysis). Free radicals and ROS like O_2_^-^, ^1^O_2_, ·OH, ·OOH, NO· and H_2_O_2 _can cause cell injury or death by oxidative stress [5,6]. Oxidative stress to the cell results from such things as DNA damage or lipid peroxidation. Disease can then develop as a direct result of radiation damage or due to a system impairment caused by radiation damage such as the case of radiation-induced damage of chromosomes in lymphocytes compromising the immune system's ability to prevent tumor development [7]. Overall, the greatest risks from radiation exposure are assumed to be cancer [8], cataracts, and damage to the central nervous system [9]. Thus the nature of the problem seems similar to nuclear systems in that systematic manifestations result from a chain of events initiated by ionization and propagated, in this case, by ensuing chemical reactions and biological responses.

Interestingly enough, oxidative stress has been implicated to play a role in the development of other diseases as well [10,11]. That is, the normal production and development of a variety of disorders and diseases has also been associated with an increase of oxidative stress and inflammation similar to that which would be caused by exposure to radiation. For example, certain detrimental effects from space radiation on the dopaminergic system are similar to functional changes that occur from Parkinson's disease [9], diabetogenic problems associated with increased C-peptide excretion and insulin resistance [12], as well as constipation due to malfunction of the intestine. Oxidative stress during space flight can cause a loss of protein after reductive remodeling of skeletal muscle due to undernutrition [13]. Diseases in which oxidative stress is implicated, and thus which could also be affected by the countermeasures proposed in this paper, include cardiovascular disease, cancer [14], chronic inflammatory disease [15], hypertension [16], ischemia/reperfusion injury [5], acute respiratory distress syndrome (ARDS) [17], neurodegenerative diseases such as Parkinson's disease and Alzheimer's disease [18,19] and aging [20].

### Radical Scavenging & Antioxidants

The actual chemical reactions that ensue and their by-products depend upon what the radicals come into contact with. For example, in pure water, radical-radical interactions lead to the formation of the decomposition products while radical-decomposition product reactions lead to the reformation of water. In a nuclear system, manifestation of system level problems has been curtailed by interfering with the chain of events early on during the chemical stages through the use of additives that alter water composition. Whereas some additives have been found to promote and increase water decomposition, others have been found to suppress it [21]. This occurs through scavenging in which the additives preferentially react with the radicals. Scavenging has the effect of removing reactive species from the system and thereby reduces their ability to participate in chemical reactions that cause decomposition. While there are various additives that preferentially react with decomposition products, the byproducts of the scavenging reaction are a factor as well since they are a component of the water composition. For example, some ionic impurities scavenge radicals (shown in 1-4) but do so at the expense of water reformation as the consumed H and OH radicals are no longer available to react with decomposition products in the reactions that lead to water reformation.

(1) $$\text{OH}+\text{B}{\text{r}}^{-}\to \text{Br}+\text{O}{\text{H}}^{-}$$

(2) $$\text{H}+\text{Br}\to \text{B}{\text{r}}^{-}+{\text{H}}^{+}$$

(3) $$\text{H}+\text{C}{\text{u}}^{++}\to \text{B}{\text{r}}^{-}+{\text{H}}^{+}$$

(4) $$\text{OH}+\text{C}{\text{u}}^{+}\to \text{C}{\text{u}}^{++}+\text{O}{\text{H}}^{-}$$

However, the use of excess H_2 _in a water system exposed to radiation provides the initial H radicals for a chain reaction that promotes water reformation and in which there is no net consumption of H_2 _in the process (shown in 5-6).

(5) $${\text{H}}_{2}+\text{OH}\to {\text{H}}_{2}\text{O}+\text{H}$$

(6) $$\text{H}+{\text{H}}_{2}{\text{O}}_{2}\to {\text{H}}_{2}\text{O}+\text{OH}$$

**Figure 1:**
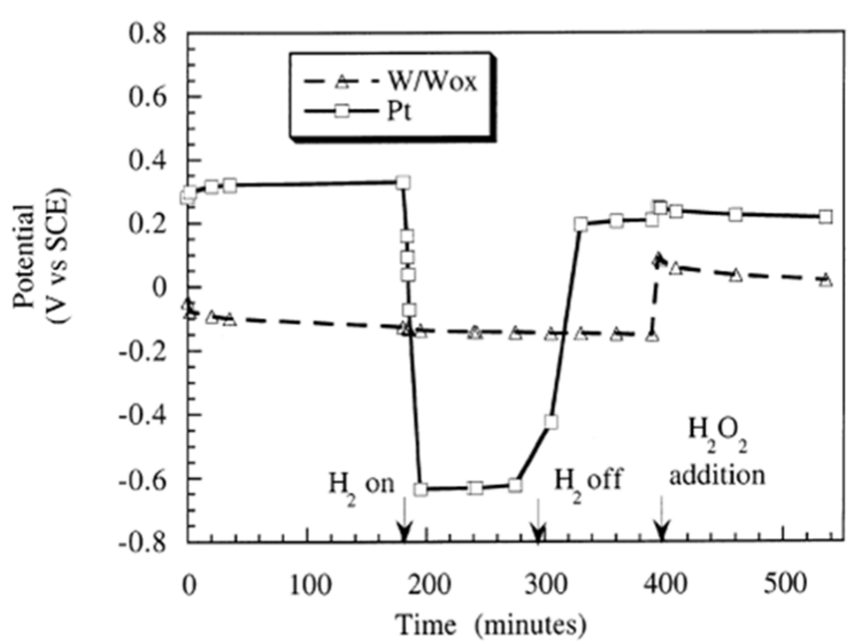
**The effect on OCP of the solution from H_2 _gas bubbled into it and the addition of 0.1 M H_2_O_2 _as measured by Tungsten/Tungsten-Oxide (ref. electrode) and Platinum electrodes (vs. saturated calomel electrode SCE) **[22].

The ability of H_2 _to suppress total oxidant concentrations in a water system exposed to radiation has long been recognized by the boiling water reactor (BWR) community and is referred to as hydrogen water chemistry (HWC). The first full-scale HWC test in the U.S. was performed at Dresden-2 in 1982 and similar tests have subsequently been carried out in several reactors [4]. In the presence of excess H_2_, both the water decomposition and production of O_2 _can be suppressed through a chain reaction which rapidly reduces the concentration of OH and H_2_O_2_. In an accelerator application, Lillard *et al. *[22] has shown that this has the effect of suppressing the Open Circuit Potential (OCP) of the water (Figure 1) and thereby electrochemically reduces the driving potential for corrosion.

Similarly, in a biological system, antioxidants have been seen to protect against oxidative stress and prevent the pathological process of a wide range of disease [23]. The effect of antioxidants in reducing oxidative stress can be attributed to their ability to protect tissues from free radicals [6] hinting towards a scavenging mechanism. Turner [24] indicates, "A number of radiosensitizing chemicals and drugs are known. Some sensitize hypoxic cells, but have little or no effect on normally aerated cells. Other agents act as radioprotectors reducing biological effectiveness...which scavenge free radicals. Still other chemicals modifiers have little effect on cell killing but substantially enhance some multistep processes, such as oncogenic cell transformation." Thus it appears that antioxidants act similarly to radical scavengers in nuclear coolant systems in that they chemically protect against indirect ionization by preferentially reacting with the reactive species and thus reducing their ability to cause oxidative stress.

**Figure 2:**
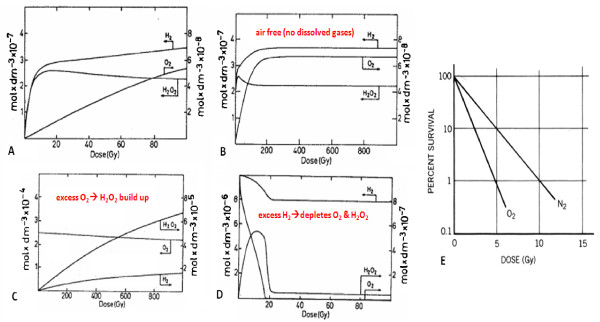
**(A)-(D)**[25]**reflect water decomposition by the concentration of radiolysis byproducts**. (B) is an extension of (A) and is air free pure water. Decomposition ensues until H_2 _in excess of ROS. (C) is effect of dissolved O_2 _in excess of H_2 _to promote decomposition. (D) is effect of dissolved hydrogen in excess of O_2 _to scavenge. (E) [24] Shows effect of O_2 _as a biological radiosensitizer. N_2 _is also shown which raises the question of what the effect of H_2 _would be.

The dependency of the outcome on scavenger type is also similar to nuclear systems where the effect of the type of additive can either be to promote water decomposition or water reformation. One such example is the effect of oxygen. There appears to be parallels in the effect of oxygen to promote water decomposition in a nuclear system and increased radiosensitivity of cells in the presence of oxygen as shown in Figure 2C[25]. With increased water decomposition, it would be expected that there would be more ROS produced leading to increased damage. This is the case as cell survival decreases under oxic conditions when exposed to X- and γ-rays implying that the indirect effect of radiolysis byproducts are the most damaging to the cell. This oxygen effect is quantified by the oxygen enhancement ratio (OER) that reflects the relative increase of radiation dose needed to produce the same biological damage under hypoxic conditions as opposed to oxic conditions. Figure 2D[25] includes the effect of hydrogen on water decomposition and shows that when in excess of ROS like O_2 _and H_2_O_2_, the water reformation process dominates as ROS are quickly scavenged. This raises the question of what would the effect of H_2 _be on radiosensitivity of cells? Also noteworthy in Figure 2 is the occurrence of an equilibrium where the amount of molecular decomposition byproducts from radiolysis remains constant. This hints at a scenario of two competing processes in which a critical point occurs when a balance is achieved between water decomposition and reformation and suggests that radical scavengers can shift which process dominates. Biological parallels and implications of this are discussed next.

### A Scenario of Competing Processes with a Critical Point & Natural Repair Mechanisms

**Figure 3:**
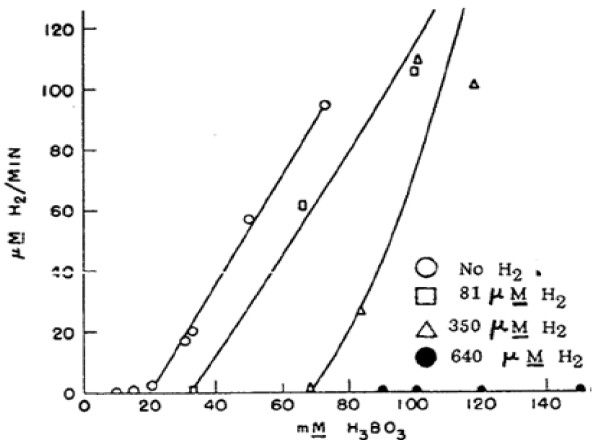
**Relative contribution of the water decomposition process is associated with boric acid concentration measured in milli-molar on the abscissa**. System scavenging capacity or relative contribution of the water reformation process is associated with the initial amount of dissolved H_2 _measured in micro-molar concentrations (each curve). Manifestation of negative systematic effects is reflected by the amount of water decomposition from radiolysis as reflected by H_2 _gas generation rates measured in micro-molar concentrations per minute on the ordinate. Figure illustrates that the addition of dissolved H_2 _increases the scavenging capacity of the water therefore increasing the threshold and delaying the onset of when decomposition becomes the dominant process [26].

Radiolysis of water can be viewed as a scenario of competing processes, water decomposition and reformation, in which the outcome will depend upon where the processes reach a balance or equilibrium. Decomposition will still occur even in the presence of additives but they serve to alter the net outcome by affecting the chemical reactions such that one process becomes more dominat. This was seen somewhat in Figure 2 and is shown more explicitly in Figure 3[26] which shows that the equilibrium point or threshold for which beyond negative effects manifest can be increased through bolstering the scavenging capacity and altering the balance such that the favorable processes are dominant.

In a biological system, it appears to be a similar scenario between biochemical damage and repair processes. Free radicals and ROS were identified as the root cause of oxidative stress and while their production is attributed to exposure to external sources like X-rays, ozone, cigarette smoke, air pollutants and industrial chemicals [27], they are also generated naturally during a variety of energy-generating biochemical reactions and cellular functions [5]. In fact, the ROS actually serve a necessary function as signaling molecules that critically modulate the activation of the immune system and thus participate in antibacterial defense [28]. Thus, neutralization of all free radicals would not be desirable. Oxidative stress occurs when there is an imbalance between antioxidants and ROS and free radicals [29] such as when ROS concentrations increase due to radiation exposure generating them by ionization. Chopping [3] observes, "The cell is protected by different DNA repair mechanisms which try to restore the damage. We don't know the details, except when the repair goes wrong (e.g. a replacement of a lost nucleotide by a 'wrong" base pair, etc.)... The cell contains natural radical scavengers. As long as they are in excess of the radiolysis products, the DNA may be protected. When the products exceed the amount of scavengers, radiation damage and cancer induction may occur. In principle, there could thus be a threshold dose for radiation damage, at which the free radicals formed exceed the capacity of scavenging. The scavenging capacity may differ from individual to individual depending on his/her physical condition." Experimental investigations regarding long-duration space flights in particular clearly showed increased oxidative stress markers and a reduction in antioxidants after these flights [30,7]. Kennedy et al. [31] demonstrated that exposure to space radiation may compromise the capacity of the host antioxidant defense system and that this adverse biological effect can be prevented, at least partially, by dietary supplementation with agents expected to have effects on antioxidant activities. Interestingly and similarly so, the radiation resistance of the bacteria *Deinococcus radiodurans *that can grow under chronic γ radiation (50 Gy/hr) or recover from acute doses greater than 10 kGy has been attributed to the role of antioxidants in mitigating the extent of oxidative damage [32-34]. Thus there appear to be similarities between the nuclear and biological systems in how use of scavengers can enhance and bolster the favorable process thereby increasing the natural radiation resistance of the system. Chopping [3] points out that several radiation protection agents are known and probably function as scavengers for the products of water radiolysis. However, the oxygen effect to promote ROS production isn't seen for the higher LET α radiation where the OER is 1, as opposed to 3 as for the case of X-rays, implying that direct damage such as double strand DNA breaks becomes the more dominant type of damage process for higher LET radiation. Therefore, for the high LET space radiation, scavenging alone may not be an effective mitigation approach. Thus, we envision a strategy that interrupts the chain of events leading to biological disease during the chemical and biological stages. In particular, we propose a strategy that (1) bolsters antioxidant capacity (2) supports natural repair processes and (3) manages biological response to radiation insult. This approach could have a great effect for increasing the threshold tolerance for radiation damage before it propagates into systematic symptoms, disease and ailments.

## Radiation protection by a conjunctive bio-chemical approach

Over the course of the last century, a wealth of knowledge has been accumulated on the effect of radiation on biological systems. Areas spanning in scope from DNA damage up to changes in physiology have received extensive study. To date, biology studies of radiation damage have largely focused on components of DNA repair systems such ataxia telangiectasia mutated gene (ATM). More recently, however, it has been found that modification of key molecular targets can protect tissue from radiation induced fibrosis in mice exposed to doses up to 25 Gy [35,36]. It has also been found that changes in APOE (Apolipoprotein E) genotype dramatically influences survival following Total Body Irradiation (TBI) in murine models. These results imply that modification of key molecular targets to induce biological changes in the host can protect tissue from radiation damage. Turner [24] notes that, "for carcinogensis or transformation, for example, such biological promoters (radioprotectors) can dwarf the effects of physical factors, such as LET and dose rate, on dose-response relationships."

Radioprotectors have been implicated to work by the following chemical and biological protective mechanisms:

1. radical scavenging of toxic decomposition products of free radicals and ROS

2. repair of biological molecules by donation of H atoms since hydrogen bonds are among the weakest in biological molecules and such are the first to be broken [37]

3. interaction with cellular components (binding, altering metabolic pathway, etc.)

Interaction with cellular components can have biological effects that lend to radioprotection like hypoxia, alteration of metabolic state, and anti-apoptotic and anti-inflammatory properties. Tissue hypoxia decreases the radiosensitivity of cells by minimizing the O_2 _effect and can be produced chemically by impairing oxygen transport (binding up hemogloblin with another molecule) or biologically by either restricting blood flow (vasoconstrictor drug, hypocapnia, etc.) or lowering blood pressure (vasodilator drug). Vasodilation along with other circulatory enhancements may also enhance the natural repair mechanism as it is believed to be more effective in a living organism, where the cells are in continuous exchange with the surrounding cells and body fluids, than in the tissue samples often studied in the laboratory [3]. Inducing a hypometabolic state which resembles hibernation, may contribute to tolerance against oxidative stress. Metabolic rates in hibernating marmots and ground squirrels help delay the onset of obvious damage. Also, survival times for guinea pigs that have received massive doses of radiation (> 6000 rads) have been extended from several hours to about 4 days through the use of central nervous system depressants (pentobarbital) where it has been attributed to partial protection from central nervous system syndrome [37]. Furthermore, a hypometabolic status may also prove to be an ideal therapy for various shock or trauma states in which dramatic reduction in metabolic demands may be highly protective [38]. Anti-apoptotic properties can mitigate organ damage such as in IR injury by reducing the amount of cellular self destruction. Interference with mitosis and DNA synthesis may slow cells in their radio-resistant phase of cell division or afford more time for natural repair of the cell prior to replication of the damage.

### 

#### We hypothesize that therapeutic medical gases can serve as radioprotectors and biological signaling molecules to work conjunctively in preventing, protecting, and repairing radiation damage

Medical gases might prove to have lower chemical toxicity and thereby permit increased dose administration. If so, this could improve effectiveness as many of the radiation protective agents are limited to being administered in small doses due to their chemical toxicity [3]. Furthermore, incorporating the biological aspect with the chemical aspect of scavenging radiolysis byproducts may prove to be particularly effective for space radiation than using low LET radioprotectors as direct damage such as DNA double strand breaks likely become the more dominant damage mechanisms for the higher LET radiation [3]. NO, CO, H_2_S and H_2 _are gaseous signaling molecules in humans. These molecules act as transmitters of information between cells by chemically interacting with cell receptors to trigger a response within the cell. These comprise some of the medical gases of interest and many of them act both on the chemical level in the form of antioxidant radical scavenging and on the biological level in the form anti-inflammatory, anti-apoptotic, and other biological effects. Extensive and more detailed information about these gases in a therapeutic role can be found in reference [23] which provides a detailed description of medical gases of interest and their properties and [39] provides detailed information pertaining in particular to H_2_.

### Hydrogen

#### We hypothesize that hydrogen can repair biological radicals by H atom donation and/or supplement antioxidant capacity either directly as an antioxidant or indirectly as a signaling molecule to trigger production of natural antioxidant enzymes

Hydrogen properties as a medical gas are summarized in Table 1. Hydrogen may have potential as a safe and potent therapeutic medical gas, as well as several potential advantages over current pharmacological therapies for the following reasons:

**Table 1:** Cited Properties of H_2 _as a Medical Gas with Suggested Chemical/Biological Mechanisms.

Biochemical Mechanism	Notes
radical scavenging antioxidant	• selectively reduces hydroxyl radicals (•OH) and reactive nitrogen oxide species (NO_2 _and N_2_O_3_) but did not eliminate O_2_^- ^or H_2_O_2 _when tested in *in vitro *[40].
	• does not decrease the steady-state levels of nitric oxide (NO) [40] which may be beneficial as endogenous NO signaling pathways modulate pulmonary vascular tone and leukocyte/endothelial interactions [64].
	• increases antioxidant enzymes such as catalase, superoxide dismutase or heme oxygenase-1 [39,44].
	• diminished lipid peroxidation as indicated by MDA levels when compared to air-treated grafts [65].
	• drinking hydrogen-containing water with concentrations as low as 0.04 mM, significantly reduced the loss of dopaminergic neurons, decreased accumulation of DNA damage, and lipid peroxidation in mice with Parkinson's disease induced by oral administration of MPTP [66].
anti-apoptotic	• postulated to inhibit caspase-3 activation [67].
anti-inflammatory	• down-regulation of pro-inflammatory cytokines, such as interleukin (IL)-1 β, IL-6, chemokine (CC motif) ligand 2 and tumor necrosis factor-α (TNF-α) [68,69].

• It is highly diffusible and as such may potentially reach subcellular compartments, such as mitochondria and nuclei, which are the primary site of ROS generation and DNA damage [40] and are also notoriously difficult to target pharmacologically.

• Its hyporeactivity with other gases at therapeutic concentrations may allow hydrogen to be administered with other therapeutic gases, including inhaled anaesthesia agents [41].

• H_2 _may spare the innate immune system while still allowing phagocytosis of infecting organisms. When tested *in vitro*, it did not eliminate O_2_^- ^or H_2_O_2 _which have important functions in neutrophils and macrophages as they must generate ROS in order to kill some types of bacteria engulfed by phagocytosis [40]. It is not clear whether a similar reaction preferentially occurs under complex biological conditions. Experimental studies have demonstrated that hydrogen has potent therapeutic efficacies on both parasite infection [42] and polymicrobial sepsis [43].

• No adverse effects have been found in humans drinking hydrogen water in a study that examined the effects of drinking hydrogen-rich water (HW) for radiation-induced late adverse effects [44,45]. Studies showed that the consumption of HW for 6 months resulted in significant decrease of serum levels of derivatives of Reactive Oxidative Metabolites (dROMs) and an increase of biological antioxidant power determined by Free Radical Analytical System (FRAS). No severe adverse effects were seen during follow up period. These results suggest that drinking HW improved Quality of Life (QOL), associated with decrease of oxidative injury markers, in patients with radiotherapy.

Hydrogen has only recently been considered for therapeutic applications for radiation exposure [46,47] and recent results are beginning to preliminarily demonstrate its radioprotective effects in cultured cells and rats when exposed to 4-8 Gy of γ-irradiation from a Co-60 source [48]. Qian, et al [48] found that a hydrogen rich PBS treatment applied to human lymphocyte AHH-1 cells increased cell vitality in that it decreased cellular lactate dehydrogenase (LDH) leakage and attenuated apoptosis. When the treatment was applied *in vivo *to male BALB/c rats, they found it attenuated intestinal injury, helped sustain levels of natural antioxidant enzymes GSH & SOD, and reduced both lipid peroxidation (as indicated by MDA) and oxidative stress (as indicated by DNA base damage/lesion 8-OHdG). The protective effects appear to be concentration dependent, at least within the range of their test (up to 0.4 mmol/L), and are more effective as a pre-treatment before exposure rather than after. This may imply a protective mechanism from an antioxidant role either by the hydrogen itself or by it 'signaling' the production of natural anti-oxidant enzymes. While there appears to be insignificant differences in levels of natural antioxidant enzymes GSH & SOD from the treatment in this experiment, other experiments have indicated hydrogen treatment appears to increase antioxidant enzymes such as catalase, SOD or heme oxygenase-1 [39,44]. None the less, a protective effect seems apparent and questions of how much hydrogen can be absorbed by ingestion, inhalation or injection and how long it will remain effective along with other questions remain to be addressed.

### Nitric Oxide

#### We hypothesize that NO and thrombospondin-1 signaling might be used conjunctively to manage response to radiation insult for tissue preservation

**Figure 4:**
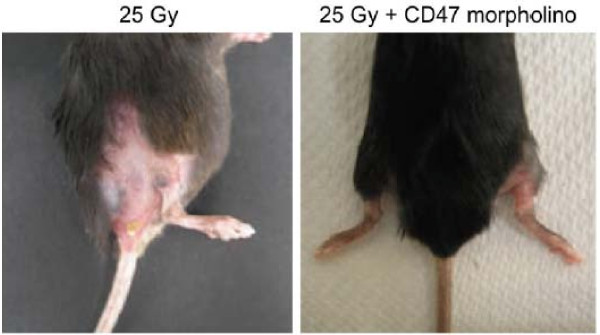
**Images compare treated and untreated hind limbs 8 weeks after an exposure of 25 Gy showing that signs of fibrotic contractures occurred only in the untreated limb**[49].

Medical properties for NO are summarized in Table 2 and effects are shown in Figure 4[49]. NO regulates platelet activity, preservation of the normal structure of the vessel wall and causes blood vessel dilation which may increase tissue blood supply [23]. This could abate inflammatory response and thus protect tissue from oxidative injury. Results from NO studies that have examined the ability of patients to inhale NO to improve outcome of acute respiratory distress syndrome (ARDS) have had discrepant results from positive, negative or neutral outcomes. Thus NO may be linked with both protective and toxic effects depending upon concentrations, source, timing of administration and the environment suggesting a narrow window for administration in the treatment of oxidative injuries [50]. Reduction of excessive and deleterious NO effects appear to be controlled by blocking NO/cGMP signaling through thrombospondin-1 signaling via its receptor CD47. This has shown to both maintain the viability of normal tissues against radiation induced fibrosis in murine models following total body irradiation (25 Gy) and increase the radiosensitivity of tumors [35,36,50].

**Table 2:** Cited Properties of NO as a Medical Gas with Suggested Chemical/Biological Mechanisms.

Biochemical Mechanism	Notes
radical scavenging antioxidant	• NO reacts with peroxy and oxy radicals generated during the process of lipid peroxidation. The reactions between NO and these ROS can terminate lipid peroxidation and protect tissues from ROS-induced injuries [70].
	• induces the rate-limiting antioxidant enzyme, heme oxygenase (HO)-1 thus imparting resistance to H_2_O_2 _induced cell death [71].
	• in bacteria, activates the redox-sentive transcriptional regulator protein (oxyR), resulting in the subsequent expression of protein protective against ROS [72].
anti-inflammatory	• inhibiting P-selectin expression and leukocyte recruitment [73].
decreased radiosensitivity	• vasodilator through relaxation of vascular tone by stimulating soluble guanylate cyclase (sGC) and increased cGMP content in vascular smooth muscle cells [23].

### Carbon Monoxide

#### We hypothesize that small, therapeutic concentrations of CO and/or when used in conjunction with other medical gases can decrease radiosensitivity without the deleterious effects of excessive CO

**Figure 5:**
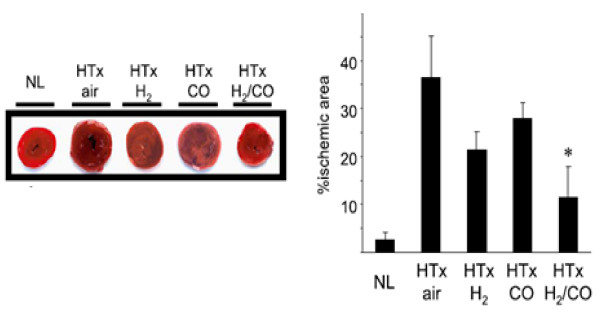
**Extent of gross structural damage to heart graft was evaluated by TTC staining 3 hr after reperfusion**. H_2 _and CO inhalation reduced ischemic area following heart grafts but with only slight significance. Significant reduction is seen by dual treatment [23].

Table 3 summarizes medical properties of CO gas. Figure 5 shows that administration of H_2_/CO mixtures has been shown to reduce structural damage to hearts in Lewis rats undergoing heart transplantation (HTx) in which oxidative stress injury is caused by ischemia/reperfusion [23] rather than radiation exposure. CO protects due to its capacity to bind hemoglobin and thereby impair oxygen transport [37] which will reduce radiosensitization caused by the O_2 _effect to promote radical production. However, when used solely, good protection is obtained when an animal has 2/3 of its hemoglobin bind in the form of carboxyhemoglobin. At this point however, the animal is in a critical state [37] and ischemic damage, metabolic acidosis and infections are potentiated. While the adverse effects of inhaled CO are a major concern for clinical use, experimental models have demonstrated that potent therapeutic efficacies exist at low concentrations [23,51]. Soluble forms of CO, such as CO-releasing molecules, may overcome the problem of tissue hypoxia and allow clinical application [52,53]. Recent animal studies have shown discrepant results between exhibiting and not exhibiting anti-inflammatory effects [23]. These discrepancies may be attributed to species specific differences in the affinity of CO for hemoglobin, or physiological differences such as respiratory rate and sensitivity to lipopolysaccharides (endotoxins) [54,55]. King and Lefer [56] point out, "When tissue is subjected to ischaemia, the lack of oxygen prevents mitochondrial respiration and oxidative phosphorlyation, which leads to a rapid decline in ATP concentration (Halestrap, 2010). Upon reperfusion, oxygen and substrates are restored to the tissue and the respiratory chain can restart, which leads to mitochondrial re-energization. This process allows mitochondria to take up Ca^2+ ^that has accumulated during ischaemia (Halestrap, 2006). However, this restoration of oxygen also causes a surge in free radicals produced by mitochondria. The combination of oxidative stress and high matrix Ca^2+ ^are ideal conditions for the induction of the mitochondrial permeability of transition pore (MPTP). The MPTP causes mitochondria to break down rather than synthesize ATP and, if unrestrained, can lead to cell death by way of necrosis." However, they continued in highlighting work from Elrod *et al. *[57] which indicates that isolated mitochondria subjected to 30 min of hypoxia, had a greater recovery of post-hypoxic respiration rate when treated with Na_2_S. As well, treating at reperfusion afforded a reduction in mitochondrial swelling and an increase in matrix density suggesting preservation of mitochondrial function [56]. This may suggest that CO treatment may be enhanced when used in conjunction with H_2_S or some sort of MPTP inhibitor like ciclosporin A [56] in which King and Lefer referenced a study by Shanmuganathan et al 2005.

**Table 3:** Cited Properties of CO as a Medical Gas with Suggested Chemical/Biological Mechanisms.

Biochemical Mechanism	Notes
radical scavenging antioxidant	• binds to the heme moiety of mitochondrial cytochrom *c *oxidase. By binding to the heme, CO may prevent degradation of heme proteins which induce tissue injury by rapidly promoting peroxidation of the lipid membranes of cells [74,75].
	• reduces mitochondria-derived ROS thus resulting in lower levels of ROS generation in which an adaptive cellular response is triggered leading to cell survival rather than cell death [76-78].
	• can induce HO-1 in cells to protect against injury [79-81]. Thus, detrimental excess of heme can be immediately removed by HO-1 enzymatic activity induced by CO.
decrease radiosensitivity	• impedes O_2 _transport as it binds to hemoglobin with an affinity 240 times higher than that of O_2_.

### Hydrogen Sulfide

#### We hypothesize that H_2_S administered in small, therapeutic concentrations can enhance antioxidant activity and aid in tissue preservation. Furthermore, it may support natural DNA repair mechanisms by temporarily slowing cell cycle progression so that more time is afforded to operate before detrimental errors are copied

The medical properties of H_2_S are summarized in Table 4. H_2_S exerts a wide range of physiological roles in mammalian tissue that contribute to cellular homeostasis and protect the cell against oxidative stress, apoptosis, and necrosis [56]. It is produced enzymatically at micromolar levels in mammals and is believed to help regulate body temperature and metabolic activity at physiological concentrations [58,59]. It has been implicated as the mechanism by which consumption of garlic attenuated cardiovascular disease where production of the gas has been demonstrated to occur by bioconversion of garlic-derived polysulfides by red blood cells [59].

**Table 4:** Cited Properties of H_2_S as a Medical Gas with Suggested Chemical/Biological Mechanisms.

Biochemical Mechanism	Notes
radical scavenging antioxidant	• antioxidant inhibitor of peroxynitrite-mediated processes via activation of N-methly-D-aspartate (NMDA) receptors [82].
	• shield cultured neurons from oxidative damage by increasing levels of glutathione [83].
	• induce upregulation of HO-1, anti-inflammatory and cytoprotective genes [84,85].
	• inhibits myeloperoxidase and destroys H_2_O_2 _[86].
	• mediates mitochondrial preservation in post hypoxic conditions that are ideal for mitochondrial permeability transition pore (MPTP) that would cause the mitochondria to break down and lead to cell death [56].
anti-apoptotic	• reduces IR induced apoptosis via reduction of cleaved caspase-3 and cleaved poly (ADP-ribose) polymerase (PARP) [87].
	• protection of isolated mitochondria by decreasing Ca^2+ ^loading via vascular smooth muscle K_ATP _channel-mediated hyperpolarization [23,38,56] or inhibition of L-type Ca^2+ ^channels.
	• H_2_S activated STAT3 and Protein Kinase C (PKC) inhibits the pro-apoptotic factor Bad and upregulated the prosurvival proteins Bcl-2 and Bcl-xl by altering phosphorylation [56].
	• H_2_S influences inactivation of pro-apoptotic pathways through survival pathway of extracellular-signal regulated kinase (ERK1/2)/mitogen-activated protein kinase (MAPK) and phosphatidylinositol 3-kinase (PI-3-kinase) [56].
anti-inflammatory	• inhibit leukocyte adherence in the rat mesenteric microcirculation during vascular inflammation [38].
decrease radiosensitivity	• transiently and reversibly inhibiting mitochondrial respiration [38].
metabolic alteration	• produces a "suspended animation-like" metabolic status with hypothermia and reduced oxygen demand in pigs (who received it intravenously) [88]. and mice (who received hydrogen sulfide via inhalation) [89,90].
	• mice breathing 80 ppm of H_2_S for 6 hr reduced heart rate, core body temperature, respiratory rate and physical activity where as blood pressure remained unchanged [56].

## Possible administration methods

Hydrogen or combinations of other medical gases could be administered to astronauts by inhalation, ingestion or injection. Inhalation could be achieved though a ventilator circuit, facemask, nasal cannula, or creating a spacesuit or spacecraft atmosphere which is composed of or contains a non-flammable gas mixture of these therapeutic medical gases. The use of Hydreliox, an exotic breathing gas mixture of 49% hydrogen, 50% helium and 1% oxygen for prevention of decompression sickness and nitrogen narcosis during very deep technical diving [60], is one example of human inhalation of hydrogen gas mixtures even though this particular mixture is suited only for deep technical diving applications. Drinking hydrogen-rich water (HW) appears to have comparable effects to hydrogen inhalation [61]. Although inhaled hydrogen gas may act more rapidly, oral intake of hydrogen-rich water is another method which may be more practical for daily life or suitable for continuous consumption in preventive or therapeutic uses. Ingestion of gas dissolved solutions may prove to be more portable, easily administered, and a safe means of delivering molecular hydrogen [62]. Gas rich water in which the gases have been dissolved could be prepared by bubbling gases into solution under pressure or other dissolution methods like swept gas diffusion. However, consideration will have to be given to loss of gas over time by dissolution and diffusion. Alternatively, some therapeutic gases such as hydrogen could be generated in solution by chemical reaction with the solution such as magnesium (Mg + 2H_2_O · Mg(OH)_2 _+ H_2_). In this case for example, a magnesium stick could be inserted into the water just prior to drinking. However, consideration will also have to be given to ingestion of the produced byproducts as well. Though oral administration is safe and convenient, hydrogen can be lost from solution by dissolution and diffusion and some hydrogen is lost in the stomach or intestine, making it difficult to control the concentration of hydrogen administrated. Administration of hydrogen via an injectable hydrogen-rich solution may allow delivery of more accurate concentrations of hydrogen [63]. This method of administration has been demonstrated for hydrogen in rats [48].

## Conclusions

We hypothesize a systems approach of using various therapeutic medical gases as chemical radioprotectors in conjunction with biological signaling molecules to disrupt the chain of events initiated by radiation exposure and interfere with pathogenesis of disease. This could have a profound positive effect as it addresses prevention, protection, and repair. This represents a novel and feasible preventative/therapeutic strategy to address radiation-induced adverse events and thus the challenge of space radiation. While more studies are warranted to apply this therapy for space travel and determine details of optimum gas mixtures and therapy administration plans, it appears that it represents a potentially novel, therapeutic, and preventative strategy that may also ameliorate symptoms for other oxidative stress related diseases as has been shown in relevant ground-based (animal) models.

## List of abbreviations

8-OHdG: 8-hydroxy-2' deoxyguanosine; APOE: Apolipoprotein E; ARDS: Acute Respiratory Distress Syndrom; ATM: ataxia telangiectasia mutated gene; BWR: Boiling Water Reactor; DNA: Deoxyribonucleic acid; dROMS: derivatives of Reactive Oxidative Metabolites; FRAS: Free Radical Analytical System; GCR: Galactic Cosmic Rays; Gy: Grey; GSH: Glutathione tripeptide; HTx: Heart Transplantation; HW: Hydrogen Water; HWC: Hydrogen Water Chemistry; HZE: High Z and Energy (Z - Atomic #); LDH: Lactate dehydrogenase; LET: Linear Energy Transfer; MDA: Malondialdehyde; MPTP: 1-mtehyl-4-phenyl-1,2,3,6-tetrahydropyridine; OCP: Open Circuit Potential; OER: Oxygen Enhancement Ratio; ppb: parts per billion; ppm: parts per million; QOL: Quality of Life; ROS: Reactive Oxygen Species; SEP: Solar Energetic Particles; SOD: Superoxide dismutase; TBI: Total Body Irradiation.

## Competing interests

The authors declare that they have no competing interests.

## Authors' contributions

MS developed the concept of using Hydrogen as a radioprotectant by noting parallels between radiation chemistry of water and radiation biology and conducting the literature review in these areas and discussing with co-authors as well as the compilation of this paper. RA provided review of the concept and provided information regarding oxidative stress from space travel and advanced methods of diagnostics. AN provided review of the concept and provided information and references regarding therapeutic uses of medical gases. DW provided review of the concept and provided information regarding NO. All authors have read and approved this manuscript.

## Authors' information

Any opinions expressed are those of the authors and do not necessarily reflect the views of NASA.
